# Cell proliferation and tissue remodeling are major determinants of cancer metabolism and the response to drugs targeting metabolism

**DOI:** 10.1186/2049-3002-2-S1-P80

**Published:** 2014-05-28

**Authors:** Elke Markert, Philip Tedeschi, Sonia Dolphi, Kim Hirshfield, Joseph Bertino, Zoltan Oltvai, Alexei Vazquez

**Affiliations:** 1 Rutgers Cancer Institute of New Jersey, New Brunswick, NJ, USA; 2 Department of Pathology, University of Pittsburgh School of Medicine, Pittsburgh, PA, USA

## Background

Alterations in cell proliferation and tissue remodeling are major hallmarks of cancer. Given that these two physiological processes require a significant investment of metabolic resources, we hypothesize that the metabolism of cancer cells is, in a first approximation, determined by the magnitude of cell proliferation and tissue remodeling.

## Materials and methods

To address this question in further detail we have analyzed *in vitro* data characterizing cancer cell lines and gene expression profiles of human cancers.

## Results

We show that the metabolism of cancer cells is strongly correlated with cell proliferation and tissue remodeling. Based on *in vitro* data, cancer cell lines align from an extreme of highly proliferating cells of relatively small size to another extreme of slowly proliferating cells with large size and mesenchymal properties [1]. Similarly, in human cancers we detect a strong negative correlation between gene signatures associated with cell proliferation and tissue remodeling [2]. The stratification of human cancers based on the cell-proliferation/ tissue-remodeling signatures results in divergence in the survival plots as significant as those obtained using current site-specific classifications. Furthermore, *in vitro* data suggest that these different subtypes have a differential sensitivity to diverse drug classes targeting metabolism [1].

## Conclusions

We conclude that human cancers can be subject to a universal cell-proliferation/tissue-remodeling classification independent of their site of origin that can guide personalized treatment targeting cancer metabolism.
